# Editorial: The role of food processing in the production and bioavailability of bio-compounds critical for boosting the immune system

**DOI:** 10.3389/fnut.2022.1011211

**Published:** 2022-09-23

**Authors:** Tianou Zhang, Ivan Salmerón, Samuel Pérez-Vega, Lourdes Morales-Oyervides, Julio Montañez

**Affiliations:** ^1^Department of Kinesiology, The University of Texas at San Antonio, San Antonio, TX, United States; ^2^School of Chemical Science, Autonomous University of Chihuahua, Chihuahua, Mexico; ^3^Department of Chemical Engineering, Autonomous University of Coahuila, Saltillo, Mexico

**Keywords:** food processing technology, bioactive compounds, anti-inflammatory, antioxidant, bioavailability, bioaccessibility

Optimum nutrition through food bioactive compounds strengthens the functions of immune cells and balances oxidative stress and systemic inflammation to decrease the risk of developing chronic metabolic diseases (1). Plant-based bioactive compounds (e.g., phytochemicals) demonstrate health benefits through their antioxidant, anti-inflammatory, and immunoregulatory effects to enhance immunity (2, 3). Food bioactives need to become available for the gut endothelium (i.e., bioaccessible) and at the site of action (i.e., bioavailable) to positively influence the immune system and overall health. Thus, the effect of food processing on bioaccessibility and bioavailability of food bioactive compounds with health benefits has raised great interest. This Research Topic assembled a variety of original research and review articles related to the transformation of solubility and delivery of bioactive compounds, and the evaluation of conventional and non-conventional food technologies for food matrix processing and their effects on keeping these compounds bioaccessible and bioavailable.

Fermentation is a conventional food technology that can increase the food polyphenols content, upregulating antioxidant and anti-inflammatory properties for the prevention of diabetes and neurodegenerative diseases. Wang et al. reviewed the effects of different food processing technologies (e.g., heating, germination, fermentation, etc.) on millet total polyphenol content (TPC) and total flavonoids content (TFC), and how changes in millet polyphenols affect the anti-diabetic effect of millet. Fermentation was reported to increase the TPC (e.g., ascorbic acid, p-coumaric acid, gallic acid, and catechol), reaching the highest value after 72 h, and to change the ratio of nutritional to anti-nutritive constituents in millets. The fermentation-induced TPC increase could be attributed to the action of microorganisms releasing “mechanically trapped” phenolic compounds (PC) from the polymeric fiber structure, and the activity of carbohydrate cleaving enzymes and β-glucosidase. Black soybean is one of the nutritious crops and is widely used in traditional medicines in Asian countries. Shabbir et al. reported that black soybean fermented with *P. acidilactici* is a promising functional food to prevent neurodegenerative diseases, such as Alzheimer's disease. Fermented black soybean inhibited inflammatory biomarkers (proteinase, protein denaturation, and lipoxygenase) and cholinesterase enzymes, and increased antioxidant capacity (FRAP, ABTS, and DPPH), anthocyanins, phenolics, flavonoids, and γ-aminobutyric acid (GABA) levels. Eight bioactive compounds were quantified, showing an increase in daidzein, genistein, glycitein, (+)-catechin, quercetin, and gallic acid in fermented samples, while a decrease in rutin and soyasaponin.

Germination, as a part of the malting process also increased the TPC and decreased nutrient interfering factors, such as phytates, tannins, and oxalic acid. Wang et al. reported that the highest TPC and TFC were found after millet flour was soaked for 10–11.8 h and fermentation at 33–38.75°C for about 36 h, with decreased phytates and tannin contents by 25 and 85%, respectively. Germinated millets also exhibited the highest TPC compared to steamed and microwaved treatments, resulting from activated cell wall-degrading enzymes to release the PC bound to non-starch polysaccharides in grain cell walls. Germination increased the antioxidant activity of millet flour extracts by elevating the total phenolics, flavonoids, and GABA content, which alleviates oxidative stress and inflammation in type 2 diabetes. In addition to that, the hypoglycemic effects of millet polyphenols could also be attributed to the delayed absorption of glucose and ameliorated spike in postprandial blood glucose through inhibiting α-amylase and α-glucosidase, decreased formation of advanced glycation end-products, and improved insulin resistance by the activation of adenosine monophosphate (AMP)-activated protein kinase pathway. Alternatively, Collins and Burrows (4) reported a new false malting method to increase oat phenolic compounds avenanthramides (AVA), modulating inflammation and immune functions in human diseases and inflammatory conditions in exercise and sports (5, 6). The false malting (conventionally malted but prevented from germination) process in oats can yield total AVA levels as high as 25- to 40-fold compared to regular oats. This new technique compensates for the relatively low AVA content in regular oat batches and allows consumers to consume threshold response levels of AVA (30–60 mg) based on daily oat consumption of 50 g of oat bran.

Physical and chemical factors, such as particle size, solvent, temperature, and extraction method, may also affect phenolic compounds' extraction yield from a plant matrix. Velázquez-Martínez et al. reported the antimicrobial activity of phenolic compounds extracted from industrial byproduct sugarcane bagasse (SCB) and found the optimum condition for the highest TPC yield was obtained using an orbital shaker for 24 h with 90% methanol as the solvent. LC-MS identified desferrioxamine b, baicalein, madecassic acid, and podototarin at different concentrations in all three SCB samples. The SCB extracts showed up to 90% and 50% growth inhibition against food poisoning bacterial strains *B. cereus* and *S. aureus*, respectively. The extracts also demonstrated a 50–80% inhibitory effect against modified yeast strains harboring mutations relevant to the Bloom and Werner syndrome (SGS1), xeroderma pigmentosum A human homolog (RAD14), and colon, ovary, or renal cancer (MLH1). The percentage of inhibition and the phenolic compound contents differed depending on the origin of the SCB sample, related to the UV radiation, nutrient components, temperature, and water stress present in the different geographical regions. These findings are promising for using SCB to obtain compounds for nutraceutical, food additive, therapeutic and anticarcinogenic agents.

Thermal processing can significantly improve or affect the bioaccessibility and bioavailability of planted-based bioactive compounds. Processes such as extrusion, frying, and cooking can improve the contents of functional food components and affect the enzymatic activities that destroy inhibitors (7, 8). Microwave heating, cooking, and moderate hot-air drying increase antioxidants bioavailability in fruits and vegetables (8). Water blanching can improve the levels of flavonoids in fruits due to the expedited release of phenolic compounds from internal cells, while heat sterilization in fruits (e.g., pear juice) produces a reduction of quercetin, kaempferol, and isorhamnetin (9). Wang et al. discussed heat treatments, such as roasting, puffing, extrusion, and parboiling effects on TPC and TFC in millet. Syringic, gallic, 4-hydroxybenzoic, ferulic, and sinapic acids increased after roasting, and ferulic and p-hydroxy acids increased after parboiling. The increase of TPC and TFC after heat treatment might be attributed to the release of bound phenolic compounds such as ferulic, caffeic, and coumaric acids. Puffed proso millet showed higher polyphenol contents but extruded finger millet demonstrated lower TPC and TFC. Heat treatments on millet polyphenol contents are determined by the heating methods used, with the highest TFC or TPC observed in roasting, followed by puffing, steaming, and extrusion (second highest for TPC). In addition, microwave treatment also caused various effects on polyphenol contents depending on the types of millets and phenolic acids, and affected the eating quality characteristics and physicochemical properties of millets.

Although polyphenols demonstrate promising anti-inflammatory and immunoregulatory properties, poor solubility in water, low bioavailability or bioaccessibility, and biotransformation by gut microbiota prevent the absorption of these bioactive compounds and delivery to the target organ and tissue. To overcome these hurdles, micro- and nano-encapsulation technology has been developed to enhance the absorption of bioactive compounds, target delivery to specific tissues, and optimize their biological effects (10, 11). Future research on food science and nutrition will aim to improve the knowledge and understanding of how conventional and non-conventional food processing technologies increase the bioavailability of critical bio-compounds through rapid release from the food matrix, improved solubility, more efficient delivery systems, and optimal structural modifications, thereby benefiting the immune system and overall health.

## Author contributions

TZ summarized the articles published in the Research Topic and wrote the editorial. IS, SP-V, LM-O, and JM reviewed and provided feedback to the editorial. All authors contributed to the article and approved the submitted version.

## Conflict of interest

The authors declare that the research was conducted in the absence of any commercial or financial relationships that could be construed as a potential conflict of interest.

## Publisher's note

All claims expressed in this article are solely those of the authors and do not necessarily represent those of their affiliated organizations, or those of the publisher, the editors and the reviewers. Any product that may be evaluated in this article, or claim that may be made by its manufacturer, is not guaranteed or endorsed by the publisher.
